# Towards a Better Understanding of the Post-Gastric Behavior of Enteric-Coated Formulations

**DOI:** 10.1007/s11095-021-03163-0

**Published:** 2022-01-18

**Authors:** Miklós Tamás Katona, Melinda Kakuk, Réka Szabó, Péter Tonka-Nagy, Krisztina Takács-Novák, Enikő Borbás

**Affiliations:** 1 Department of Pharmaceutical Chemistry, Semmelweis University, 7 Hőgyes Endre Street, Budapest, H-1092 Hungary; 2 Egis Pharmaceuticals PLC, 116-120 Bökényföldi Street, Budapest, H-1165 Hungary; 3 Department of Pharmaceutics, Semmelweis University, 9 Hőgyes Endre Street, Budapest, H-1092 Hungary; 4 Department of Organic Chemistry and Technology, Budapest University of Technology and Economics, 3 Műegyetem rakpart, Budapest, H-1111 Hungary

**Keywords:** acetylsalicylic acid, biorelevant dissolution, enteric coating, gastric residence time, gastrointestinal pH

## Abstract

**Purpose:**

The aim of our work was to develop a biorelevant dissolution method for a better understanding of the *in vivo* performance of delayed-release tablet formulations.

**Methods:**

The typical pH profile and residence times in the stomach and small intestine were determined in fasted conditions based on the published results of swallowable monitoring devices. Then, a multi-stage pH shift dissolution method was developed by adding different amounts of phosphate-based buffer solutions to the initial hydrochloric acid solution. Because of the highly variable *in vivo* residence times in the stomach, two alternatives of the method were applied, modeling rapid and slow gastric emptying as well. This approach provided an opportunity to study the effect of the acidic treatment on post gastric release. Six enteric-coated low-dose acetylsalicylic acid (ASA) formulations including the reference Aspirin Protect were tested as a model compound. Moreover, the thickness of the coating of each formulation was investigated by scanning electron microscope.

**Results:**

Comparing the *in vitro* results to the known properties of the formulations, the new method was found to be more discriminative than the USP dissolution method. Ingredients affecting the *in vitro* dissolution, and thus probably the *in vivo* performance, were identified in both the tablet core and the coating of the tested formulations. The limited available *in vivo* data also indicated an increased predictivity.

**Conclusion:**

Overall, the presented method may be an efficient tool to support the development of enteric coated generic formulations.

**Graphical abstract:**

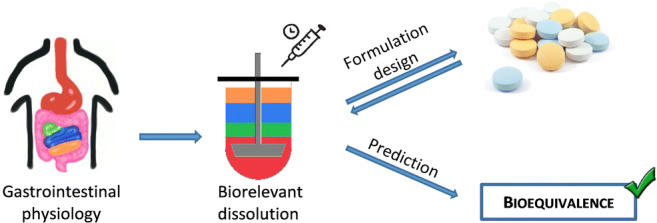

## Introduction

The reliable prediction of *in vivo* performance of generic formulation candidates is a continuous challenge in drug development. In cases where solubility or the dissolution of the API is the rate limiting factor of absorption, the *in vitro* dissolution test is the primary tool for the prediction of bioavailability (1). However, conventionally used apparatuses and buffer compositions are usually not suitable to model the complex atmosphere of the gastrointestinal system. The lack of predictive *in vitro* dissolution methods is particularly common in formulations such as enteric-coated(EC) products, which was underlined by Al-Gousous*et al*. (2).

The dissolution testing of delayed-release products for quality control (QC) purposes is specified by various Pharmacopoeias. In order to demonstrate the resistance of the coating to the gastric fluid, both EMA and FDA prescribes the testing of the product in an acidic medium (e.g. 0.1 M hydrochloric acid) for 1 to 2 h, which is followed by testing in a buffer solution of pH 6.8 to model the small intestinal environment (3, 4). As evident from the prescriptions of the Pharmacopoeias, these methods apply only one pH to model the small intestine, which is not sufficient to determine the exact site of disintegration and absorption. In general, dissolution methods for QC purposes need to be robust and simple to implement, which limits the *in vivo* predictability of the method. However, in case the aim is to support the formulation development, dissolution methods should be as biorelevant as possible in order to design the formulation able to behave *in vivo* as intended.

The aim of the application of enteric coating is to delay the release of the drug substance until it is emptied from the stomach. Thereafter, the site of drug release is affected by several factors, such as the structure of the employed film former, the thickness of the applied film, and the nature and quantities of the additives used together with it (5). In general, enteric coatings are weakly acidic polymers that are insoluble at gastric pH but ionize and dissolve under intestinal conditions. Different polymers have different pH thresholds, which is an important property when targeting the site of disintegration (2). Due to the acidic nature of coatings, the accumulation of protons on the surface of such formulations in the stomach may also affect their post-gastric release. Based on this, the residence time in the stomach also plays an important role in the subsequent absorption of the drug substance (6). Physiologically, the gastric emptying is related to the migrating motor complex (MMC) of the stomach, which is a ~2 h long cycle that consists of four phases. Phase I is a period of motor quiescence lasting 40–60% of the cycle. Phase II, accounting for 20–30% of the cycle, exhibits irregular phasic contractions. Phase III is a 5- to 10-min period of lumenally occlusive, rhythmic contractions occurring at the slow-wave frequency. Phase IV is a transitional period of irregular contractions between phase III and phase I (7). Non-disintegrating solid dosage forms administered in the fasted state are mainly emptied during the intense contractions of phase III, also known as the ‘housekeeper wave’ (8, 9). However, Kaniwaka *et al*. found significant correlation between the gastric emptying rates and the size of enteric-coated tablets as well (10).

To develop a predictive *in vitro* dissolution method, the appropriate characterization of the pH conditions and the residence times in each relevant part of the gastrointestinal (GI) system is essential. Some studies focused on the evaluation of gastrointestinal pH conditions using ingestible radiotelemetry capsules as early as the late 1980s (11, 12). To date, a number of similar, new devices have become available (e.g. Bravo capsule, IntelliCap, SmartPill) which help in the precise characterization of GI pH values (13–17). Previously, the determination of residence times in the GI tract was carried out using formulations labeled with radionucleotides (18). However, with the advance of radiotelemetry capsules, a suitable alternative is provided for this purpose as well (12, 15).

The gastrointestinal environment is strongly affected by the food and liquid intake, therefore the *in vivo* bioavailability (BA) studies are conducted under standardized conditions. According to the EMA’s guideline, in general, a bioequivalence (BE) study should be conducted under fasting conditions, as this is considered to be the most sensitive condition to detect a potential difference between formulations (19). In order to prove bioequivalence, performing a study in fasted state is prescribed by the FDA as well (20). In general, subjects are fasting for 8 h prior to administration, then test and reference products are administered with a standardized amount of water (at least 150 mL). No food intake is allowed for at least 4 h post-dose. As prescribed by the EMA, the sampling schedule of a bioequivalence study should include frequent sampling around the predicted t_max_ to provide a reliable estimation of peak exposure (19). However, for enteric-coated formulations, the high variability of gastric emptying rate results in high variability of t_max_ value, thus adequately describing their plasma concentration-time profile is challenging. This occurs especially when the plasma half-life of the investigated drug substance is short. The difficulty of *in vivo* testing of EC formulations also confirms the importance of proper *in vitro* characterization.

Acetylsalicylic acid (ASA) is a nonsteroidal anti-inflammatory drug (NSAID) which is commonly used to reduce pain, fever or inflammation (21). ASA irreversibly inhibits platelet aggregation by inhibiting thromboxane A_2_ (TxA_2_) synthesis, therefore it is also recommended in single and dual antiplatelet therapy. It has been shown that the use of ASA increases the risk of gastrointestinal bleeding especially when used long-term(22). The adverse effect is dose-related therefore in the antiplatelet indication it is typically given in low-dose (50–100 mg/day) (23). In order to avoid the irritation of the stomach, ASA is available as enteric-coated dosage form as well. Since there have been several reported attempts that failed to demonstrate BE in case of generic enteric-coated ASA formulations this substance was chosen as a model compound in our study (24–26).

According to Garbacz *et al.*, the bicarbonate buffer can be considered as the most biorelevant buffer system for the simulation of intestinal conditions. However, the disadvantage of such buffer solutions is their thermodynamic instability, which requires the control of the pH during dissolution testing (27). Despite their complicated implementation, there are a number of examples of using bicarbonate buffers for the testing of enteric-coated formulations as well (28–30). Alternatively, Al-Gousous*et al.* have successfully developed a dissolution method using phosphate-based surrogate buffer and found good correlation between *in vitro* and *in vivo* properties of Aspirin Protect 300 mg and Walgreens Aspirin 325 mg formulations. The published dissolution method considers the pH change after emptying the stomach and applies two different phosphate buffers to model the pH and buffer molarity gradient along the small intestine (31). However, the referred methods pay less attention to the effect of gastric emptying time on the performance of enteric coatings. In addition, the results of the advanced radiotelemetry capsules allow a more accurate simulation of the characteristic pH profile and residence times of the small intestine, giving a new opportunity to predict the site of disintegration and absorption.

The aim of our work was to develop a new biorelevant *in vitro* dissolution method for enteric-coated formulations considering the physiological conditions of the stomach and the small intestine, such as typical pH profile, residence times and biofluid volume. The accurate modeling of these parameters is expected to provide us with a better understanding of the site of disintegration and the rate of absorption of enteric-coated formulations. However, due to the complex composition of biofluids (enzymes, bile acids, etc.), some simplifications had to be made, to get a better applicable method.

Two alternatives of the new method, modeling rapid and slow gastric emptying, and the USP method were used to test different enteric-coated, low-dose ASA formulations. The tested formulations included the reference product as well as the commercially available generic alternatives in Hungary. Since the comparison of different enteric coatings was also aimed and each of the latter formulations contained the same type of coating polymer, Walgreens Aspirin 81 mg marketed in the USA was also tested despite having a different strength. The coatings of each formulation were examined with scanning electron microscope (SEM). The thickness and the composition of the coatings as well as the composition of the tablet cores were studied to interpret the obtained *in vitro* dissolution results. In case published *in vivo* results were available, the IVIV relationship between the dissolution profiles and the corresponding pharmacokinetic parameters was also investigated. For other formulations, the possible *in vivo* effects of the *in vitro* dissolution results were discussed.

## Materials and Methods

### Materials

Six commercially available enteric-coated ASA-containing products were tested: Walgreens Aspirin 81 mg (LNK, USA; Lot: P106919), Aspirin Protect 100 mg (Bayer AG, Germany; Lot: BTAH3CO), Asatrin-Teva Protect 100 mg (Teva Pharmaceutical Industries Zrt., Hungary; Lot: R43739), ASA Krka 100 mg (KRKA, Slovenia; Lot: D66849), Asactal 100 mg (Actavis Group PTCehf., Iceland; Lot: 037018) and ASA Protect Pharmavit 100 mg (PharmaSwiss Ceska Republika, Chech Republic; Lot: 7E126A). Walgreens Aspirin was purchased in the USA, while other products were purchased from pharmacies in Hungary. All formulations were white colored, round, cylindrical biconvex tablets with slight differences in the sizes: the height and the diameter of the formulations varied between 3 and 4 mm and 6.5–8 mm. The tested products and the inactive ingredients of the tablet cores and the applied coating materials are summarized in Table I.

**Table I:** Dose and Qualitative Ingredients of the Tested Products

Product	Dose (mg)	Core	Coating
Aspirin Protect	100	Microcrystalline cellulose, corn starch	Methacrylic acid− ethyl acrylate 1:1 copolymer, polysorbate 80, sodium lauryl sulfate, triethyl citrate, talc
Asatrin- Teva Protect	100	Microcrystalline cellulose, potato starch, silica colloidal anhydrous, lactose monohydrate	Methacrylic acid− ethyl acrylate 1:1 copolymer, triacetin, talc
ASA Krka	100	Microcrystalline cellulose, potato starch, silica colloidal anhydrous, lactose monohydrate	Methacrylic acid− ethyl acrylate 1:1 copolymer, polysorbate 80, sodium lauryl sulfate, triacetin, talc
Asactal	100	Microcrystalline cellulose, corn starch, silica colloidal anhydrous, stearic acid	Methacrylic acid− ethyl acrylate 1:1 copolymer, polysorbate 80, sodium lauryl sulfate, triethyl citrate, talc
ASA Protect Pharmavit	100	Microcrystalline cellulose, potato starch, silica colloidal anhydrous, lactose monohydrate,	Methacrylic acid− ethyl acrylate 1:1 copolymer, triacetin, talc
Walgreens Aspirin	81	Microcrystalline cellulose, corn starch, silica colloidal anhydrous, polydextrose, sodium bicarbonate	Hypromellose, methacrylic acid, shellac wax, sodium lauryl sulfate, polyethylene glycol, simecthicone, triacetin, triethyl citrate, talc, titanium dioxide

All chemicals used were of analytical grade. Sodium dihydrogen phosphate monohydrate; trisodium phosphate; acetonitrile; hydrochloric acid; (Molar Chemicals Ltd., Budapest), disodium hydrogen phosphate dihydrate; (Thomasker, Budapest), phosphoric acid; (Emsure ACS. Reag. Ph. Eur., Budapest).

### Methods

#### Dissolution Testing

The dissolution tests were carried out using an Agilent 708 DS dissolution apparatus (Agilent Technologies, Inc., Santa Clara, California, USA). The media were thermostated at 37 ± 0.5°C. Each formulation in each method was tested on six parallel samples.

##### USP Dissolution Method (32)

The samples were first placed into USP I baskets and stirred at 100 rpm in 1000 mL of 0.1 M HCl solution for 120 min, then the medium was replaced by 900 mL of pH 6.8 ± 0.5 phosphate buffer and the test was continued for an additional 60 min at constant stirring rate. The buffer solution was prepared by mixing 0.1 M HCl with 0.2 M tribasic sodium phosphate (3:1). In case it was necessary, the pH was adjusted with 2 M hydrochloric acid or 2 M sodium hydroxide. Samples at each sampling time point were taken into HPLC vials via autosampling. The sampling cannulas were equipped with 10 μm PVDF, full-flow filter tips (Agilent Technologies, Inc., Santa Clara, California, USA). The applied sampling schedule is shown in Table II.

**Table II:** Sampling Time Points of USP Method

Medium	Sampling time (min)
0.1 M HCl	60, 120
pH 6.8 phosphate buffer	5, 15, 30, 45, 60

##### Biorelevant Dissolution Method

The dissolution apparatus was equipped with 250 mL small volume vessels and rotating paddles according to Chinese Pharmacopoeia. The media were stirred at 50 rpm. The initial dissolution medium was 160 mL of 0.01 M HCl solution, which was modified in three steps through the addition of different amounts of Na_2_HPO_4_ buffer in order to simulate the conditions of the stomach and different parts of the small intestine. The addition of the buffer solution was performed using Cole Parmer 74,900 infusion pumps (Cole Parmer, Vernon Hills, Illinois, USA), the pH of the media was measured by an Inolab-type pH meter (WTW GmbH, Weilheim, Germany). Due to the highly variable *in vivo* residence times in the stomach, two variants of the method were applied which differed only in the length of the acidic treatment. The conditions of the method modeling *rapid* gastric emptying (RGE) and *slow* gastric emptying (SGE) are summarized in Table III.

**Table III:** Applied Conditions of Dissolution Method with RGE and SGE

			Method with RGE	Method with SGE
	Medium	pH	Residence time (min)	
Gastric phase	160 mL, 0.01 M HCl solution	2.0	**20**	**120**
*pH change 1.*	*Addition of 20 mL, 135 mM Na*_*2*_*HPO*_*4*_ *buffer*		*10*	*10*
Duodenal phase	180 mL, 15 mM phosphate buffer	6.5	30	30
*pH change 2.*	*Addition of 10 mL, 100 mM Na*_*2*_*HPO*_*4*_ *buffer*		*20*	*20*
Jejunal phase	190 mL, 19.5 mM phosphate buffer	6.8	70	70
*pH change 3.*	*Addition of 20 mL, 100 mM Na*_*2*_*HPO*_*4*_ *buffer*		*10*	*10*
Ileal phase	210 mL, 27.1 mM phosphate buffer	7.2	45	45

The main difference between the two method was highlighted with bold entries

Samples at each time point were taken manually using equivalent filtration to that of the USP method. The volume of the samples was 1 mL in all cases. Table IV shows the sampling schedule of the biorelevant methods.

**Table IV:** Sampling Time Points of Biorelevant Methods

Method	Sampling time (min)
RGE	20, 30, 60, 70, 80, 90, 100, 110, 120, 135, 150, 160, 175, 190, 205
SGE	120, 130, 160, 170, 180, 190, 200, 210, 220, 235, 250, 260, 275, 290, 305

Onset of the dissolution was determined by 5% of dissolved drug substance. The f_2_ statistic was calculated based on the EMA guideline on the Investigation of Bioquivalence (33).

#### Determination of Dissolved Drug Content by High Performance Liquid Chromatography

A Waters Acquity UPLC device (Waters, Milford, Massachusetts, USA) was used to determine the amount of dissolved drug in the solutions. For this purpose, YMC-Pack Pro C18 RS S-5 μm, 8 nm 150 × 4.6 mm I.D type HPLC column was used. The mobile phase was ACN:H_2_O:cc.H_3_PO_4_ = 400:600:1 and the flow rate was 1.0 mL/min. The mode of separation was isocratic. External calibration was applied by five consecutive injections of the standard solution containing the concentration of API corresponding to the approximated concentration of 100% dissolution. The calibration was controlled by the injection of the standard control solution containing the same nominal concentration, then followed by the injection of the sample solutions. The absorbance was detected at 237 nm. For standard preparations, accurate measurements were achieved using a Mettler Toledo XP26 microanalytical balance (Mettler Toledo, Columbus, Ohio, USA). The sample concentrations in mg/L were calculated using the dilution of the standard solution and the sample solution and the peak areas of the sample solutions. The chromatographic conditions for each test preparation were the same as well as the column used for the measurement.

#### Examination of Coatings by Scanning Electron Microscope

Before the test, the samples were fixed with double-sided carbon glue to copper stumps, then gilded with a JEOL 1200 type device (JEOL, Akishima, Tokyo, Japan). Images were taken from the samples in tablet form using a JEOL JSM-6380LA scanning electron microscope (JEOL, Akishima, Tokyo, Japan) applying 15 kV accelerating voltage and 10 mm sample distance under high vacuum.

## Results and Discussion

### Dissolution Results Obtained by USP Method

**Fig. 1:**
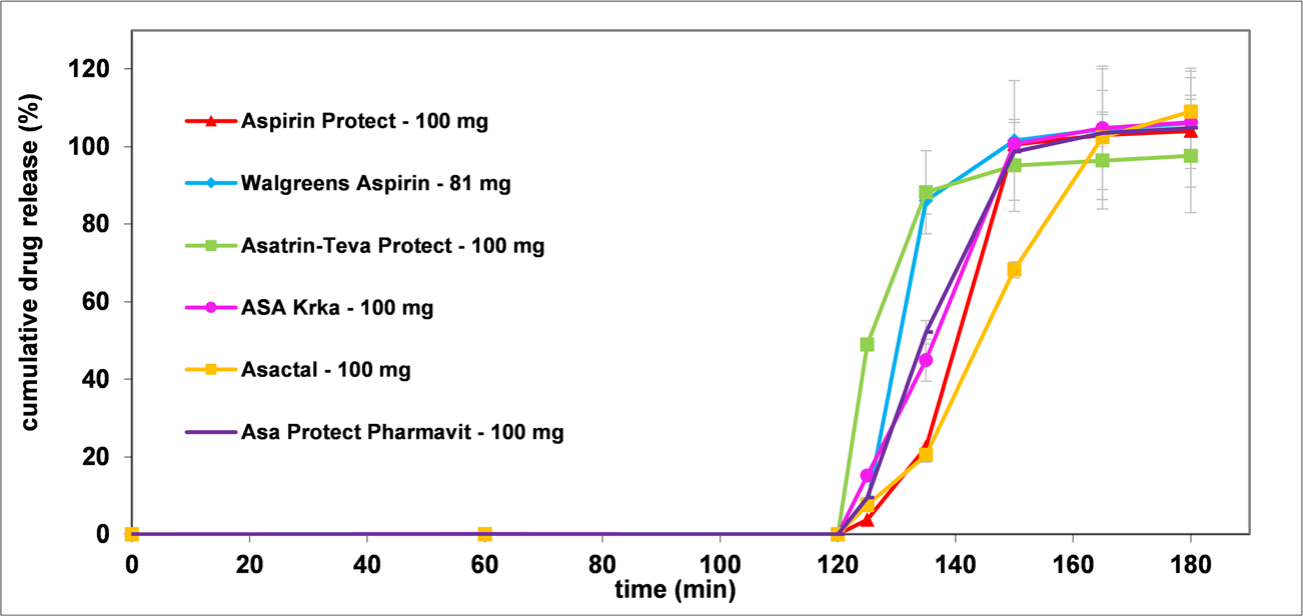
Dissolution results obtained by USP method.

A comparative dissolution study of the selected formulations was performed according to the pharmacopoeial prescriptions (32). As evident from Fig. 1, no dissolution was observed during the 2-h treatment in 0.1 M HCl solution. After the replacement of the dissolution medium by pH 6.8 phosphate buffer, each formulation started to dissolve immediately, and a measurable concentration of ASA was observed at the 5-min sampling point in all cases. The post-acidic dissolution of all formulations except Asactal was rapid (≥85% for the mean percent dissolved in ≤30 min). Walgreens Aspirin and Asatrin Teva Protect even met the criterion of very rapid dissolution (≥85% for the mean percent dissolved in ≤15 min in this medium) (34). The dissolution rate of Asactal was significantly lower as its mean dissolution exceeded 85% only after 45 min residence in pH 6.8 buffer.

These results are in agreement with the expectation for delayed release formulations that are designed to release the active substance after the dosage form has reached the small intestine, therefore do not dissolve in acidic media. As the dosage form is placed in the higher-pH environment, the polymer coating dissolves, and the tablet core behaves similarly to immediate-release formulations. Based on the results of the acid phase, the gastro resistance of the enteric coating of each formulation was found to be appropriate. As the pH 6.8 used after the pH change is typical for the *jejunum* in fasted state, the formulations are expected to dissolve in this intestinal tract at the latest. However, in the absence of a medium modeling the duodenal pH, the results do not provide information about the exact site of the onset of the drug release.

According to the individual USP monograph of Aspirin Delayed-Release Tablets not more than 10% of the labeled amount of aspirin is allowed to dissolve in the acidic stage while the dissolution in the buffer stage must exceed 75% in 45 min (35). These criteria are consistent with both USP and Ph. Eur general prescriptions for delayed-release formulations (36, 37). Based on Fig. 1, it can be determined that all formulations met the acceptance criteria. However, the *in vivo* studies performed did not demonstrate bioequivalence for either ASA Krka or Asactal formulations (24, 25). The latter also points out the importance of an appropriate biorelevant dissolution method during generic formulation development phase.

### Development of a Biorelevant Dissolution Method

**Fig. 2:**
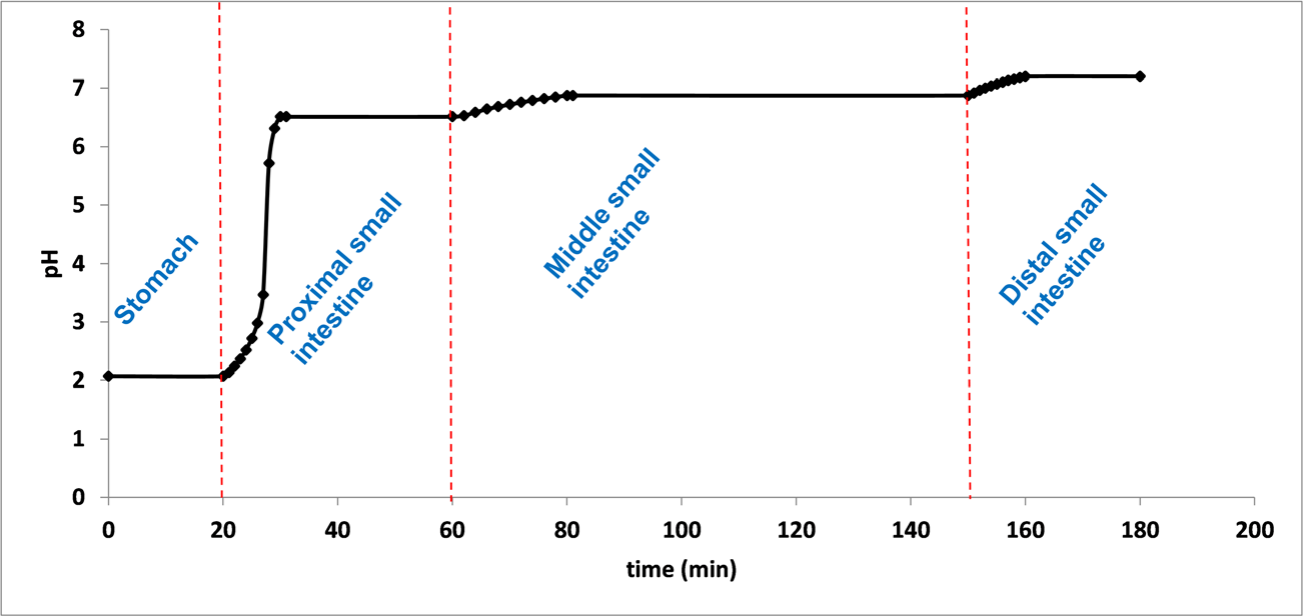
Experimental pH profile of biorelevant dissolution method with RGE.

The development of the method was focused on modeling the gastrointestinal conditions in fasted state. The applied pH conditions and residence times were determined based on published experimental results of ingestible pH monitoring capsules (11, 12, 14–18, 38). In case of such devices, gastric residence and small intestinal transit times are determined based on characteristic pH changes. As the capsule passes through the *pylorus*, the acidic environment of the fasted stomach is rapidly and sustainly replaced by an almost neutral pH of the *duodenum*. The small intestinal residence ends with the passage through the *ileocecal* valve, which is indicated by a > 0.5 decrease of pH as a result of bacterial digestion products in the colon. According to the published data, the mean pH of the stomach was found to be around 2.0, which is resulted by the dilution of the initial gastric acid with the liquid intake following the administration of the drug product. The residence time in the stomach is reported to be typically between 20 and 120 min with high variability. Since the time spent in the acidic medium may affect the physicochemical properties of the weakly acidic film formers, instead of specifying the average residence time, two versions of the method were tested, one with 20 min and one with 120 min acidic treatment, to model both faster and slower gastric emptying. The pH conditions modeling the small intestinal tracts were set to pH 6.5 (proximal phase), pH 6.8 (middle phase) and pH 7.2 (distal phase), respectively. The time spent at each pH was 30 min (proximal phase), 70 min (middle phase), and 45 min (distal phase), excluding the time of the pH changes. According to the results of radiotelemetry capsules, the pH change between each tract is rather gradual than momentary (13). To model this phenomenon, the buffer solutions were administered using an infusion pump. The experimental pH *vs* time profile of the developed method with rapid gastric emptying is shown in Fig. 2.

The composition of the gastric buffer was 0.01 M HCl solution, while the appropriate pH changes were achieved by the addition of different amounts of Na_2_HPO_4_ solutions with different molarities. The molarity of the phosphate-based buffer solutions was set based on the results of Al-Gousous*et al.*, who elaborated a simplified alternative to unstable bicarbonate buffer systems (34). The volume of the dissolution media varied from 160 mL to 210 mL, which better suits the amount of fluid in the stomach after the intake of drugs with a glass of water.

### Dissolution Results Obtained by Biorelevant Method with Rapid Gastric Emptying (RGE)

**Fig. 3:**
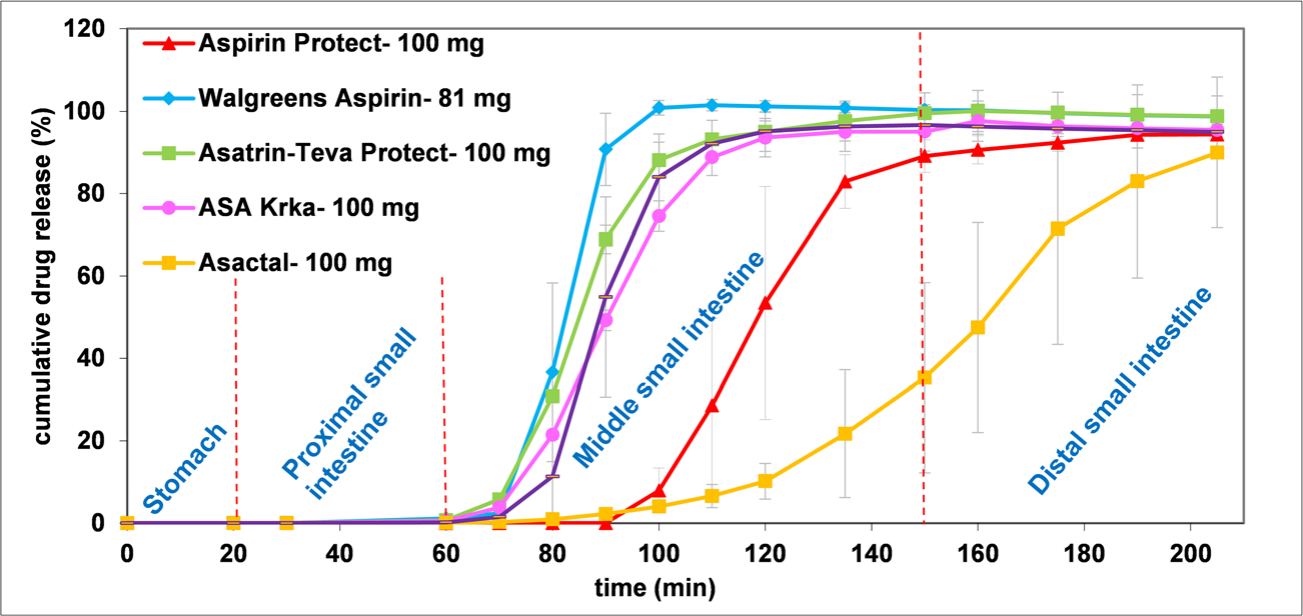
Dissolution results obtained by biorelevant dissolution method with RGE.

Figure 3 shows the results of the dissolution method modeling rapid gastric emptying. According to the results none of the products releases the API in the gastric or proximal small intestinal phase (0–60 min). In case of Walgreens Aspirin, Asatrin-Teva Protect, ASA Krka and ASA Protect Pharmavit the mean onset of dissolution ranged from 78.3 ± 4.1 to 80.0 ± 6.3 min, which belongs to the pH change between the proximal and middle small intestinal phase. The dissolution profiles of the latter formulations except Walgreens Aspirin were found to be similar, as the calculated similarity factors (f_2_) were ≥ 50 (f_2,Asatrin-Teva Protect *vs*. ASA Protect Pharmavit_ = 50; f_2,ASA Krka *vs*. ASA Protect Pharmavit_ = 58). Aspirin Protect and Asactal started to dissolve at the pH of the middle small intestinal phase (pH 6.8; 80–150 min), however, the dissolution rate of Asactal is significantly slower compared to Aspirin Protect (f_2_ = 21). The dissolution of the products except Asactal is completed or almost completed in the 80–150-min interval, while Asactal releases its API mostly in the distal small intestine.

The examined products except Walgreens Aspirin are coated with methacrylic acid − ethyl acrylate 1:1 copolymer, with a dissolution pH threshold of 5.5, which is considered to target the onset of release to the *duodenum*(2). Interestingly, the results showed that the release of the drug substance is more typical in the later small intestinal phases. The dissolution profiles of the generic formulations were different (f_2_ < 50) from that of the reference Aspirin Protect despite the same coating material, which indicated that other properties of the coating or the composition of the tablet core may also affect the release of the drug substance.

### Dissolution Results Obtained by Biorelevant Method with Slow Gastric Emptying (SGE)

**Fig. 4:**
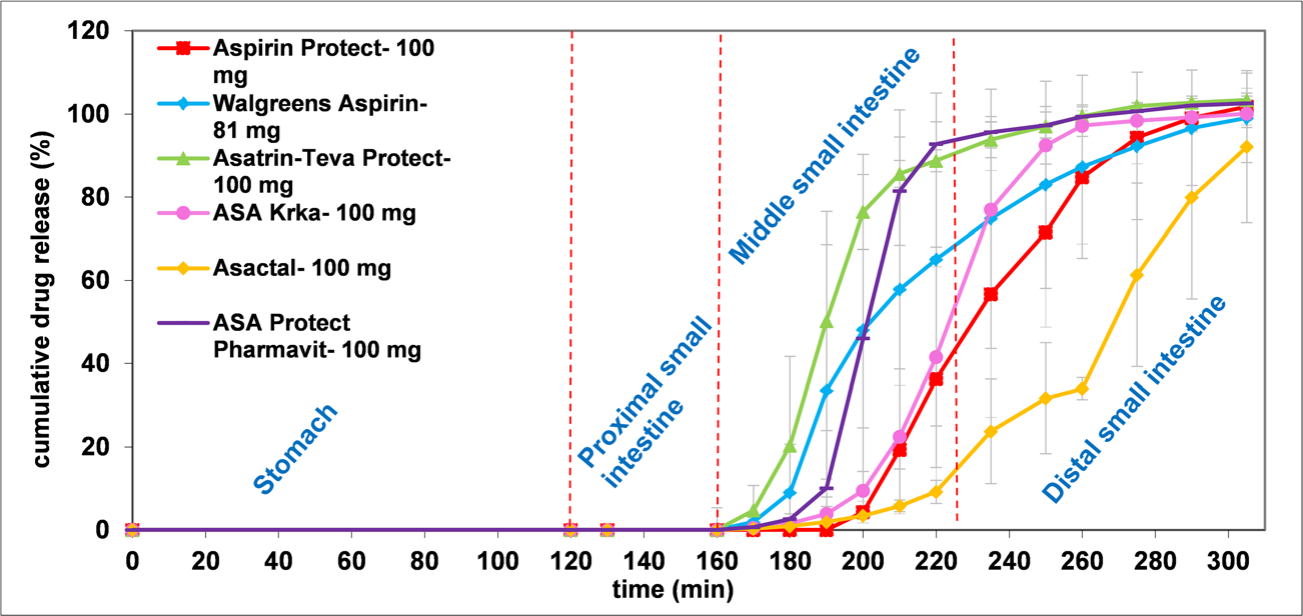
Dissolution results obtained by biorelevant dissolution method with SGE.

Figure 4 shows the results of the dissolution method modeling slow gastric emptying. Similar to the RGE method, there was no dissolution observed in the gastric and proximal small intestinal periods (from 0 to 160 min on Fig. 4). The dissolution of the formulations except Asatrin Teva Protect and Walgreens Aspirin started at the pH 6.8 period (180–250 min). In case of Asatrin Teva Protect and Walgreens Aspirin, a certain amount of API has already been released at the pH change between the proximal and middle small intestinal phases (160–180 min), however it was also less than that of the RGE method. It is also evident from Fig. 4 that, compared to other formulations, the longer gastric residence time had a greater effect on the shape of the dissolution profile of Walgreens Aspirin and resulted in longer saturation time. The mean post-gastric onset of ASA Krka dissolution was delayed by 20.0 min, while other formulations changed slightly by 3.7 to 8.3 min. Similar to the RGE method, the dissolution of Asactal is significantly slower than the reference formulation (f_2,Asactal *vs*. Aspirin Protect_ = 26) and most of the API release occurs in the simulated distal small intestine.

The observed delay in the disintegration of all formulations compared to the RGE method is most probably due to the additional accumulation of protons on the surface of enteric coatings during the longer acidic treatment. The unexpected performance of ASA Krka compared to other formulations with the same coating material requires further investigation. The results suggest that the coating material of Walgreens Aspirin is more sensitive for the longer gastric residence than methacrylic acid−ethyl acrylate 1:1 copolymer.

### Scanning Electron Microscopic Images

**Fig. 5:**
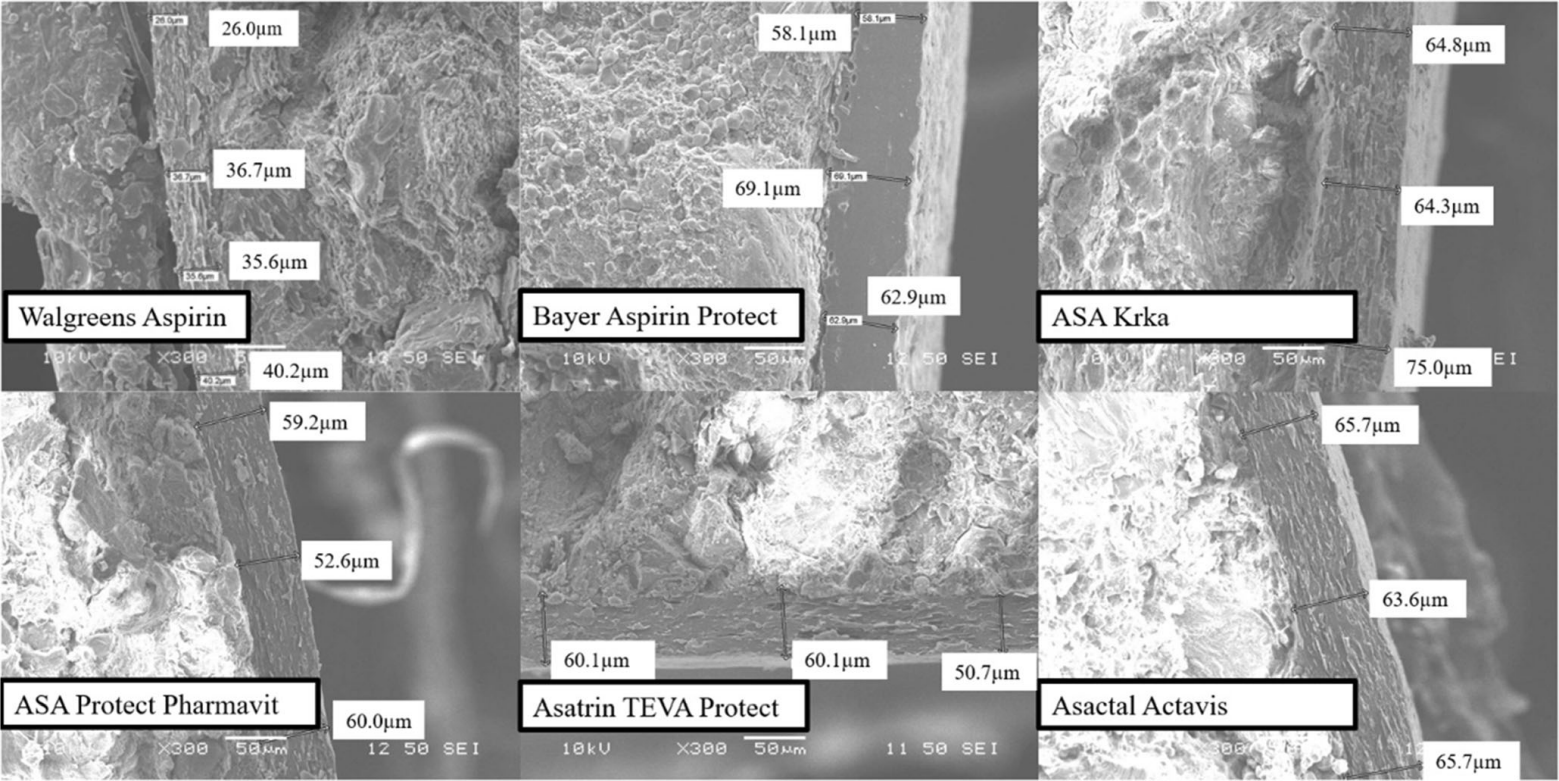
SEM pictures of enteric coated ASA formulations.

The structure and thickness of the coatings surrounding the tablet cores are shown in Fig. 5.

The coatings were found to be evenly distributed around the cores in all cases. Comparing the structure of the coating around Walgreens Aspirin with other formulations coated with methacrylic acid-ethyl acrylate, it can be said that methacrylic acid-ethyl acrylate is more concise, especially in the case of Bayer Aspirin Protect.

As evident from Table V, the coating of Walgreens Aspirin is thinner than that of other formulations. This observation is in accordance with the dissolution results of the RGE method (Fig. 3), where Walgreens Aspirin showed the highest dissolution rate.

**Table V:** Thickness of the Coating of ASA Formulations

Product	Thickness of the coating (μm)
Walgreens Aspirin	26.0–40.2
ASA Protect Pharmavit	52.6–60.0
Bayer Aspirin Protect	58.1–69.1
Asatrin- Teva Protect	50.7–60.1
ASA Krka	64.3–75.0
Asactal Actavis	63.6–65.7

In case of all five methacrylic acid-ethyl acrylate coated formulations, the thickness of coating is between 50 and 75 μm which indicates that the differences in their dissolution profiles are most probably due to other factors than the thickness of the coating.

### Relationship Between the Composition and *In Vitro*/*In Vivo* Performance

Among the tested formulations the manufacturer of ASA Krka and Asactal submitted BE study results to support the application for marketing authorization (MPA, 2016a; MPA, 2011). Other applicants, such as Teva, referred to the well-established clinical use and provided only an overview of literature references (26). A summary of the available clinical results is presented in Table VI.

**Table VI:** *In Vivo* Results of Available Clinical Studies in Fasting Conditions

Test formulation	Reference formulation	Study ID	PK parameter	Test/ref ratio
ASA Krka 100 mg	Aspirin Protect 100 mg	091B13	C_max_	1.16 (CI 0.92–1.48)
			AUC	1.16
Asactal 100 mg	Aspirin Protect 100 mg	1267/07	C_max_	1.22 (CI 1.03–1.43)
			AUC	1.18 (CI 1.05–1.33)
		1321/07	C_max_	0.76 (CI 0.65–0.89)
			AUC	0.98 (CI 0.88–1.10)
		1747/08	C_max_	1.24 (CI 1.04–1.47)
			AUC	1.12 (CI 0.97–1.29)

Actavis has performed three *in vivo* studies under fasted state to compare Asactal and Aspirin Protect, each of which failed to demonstrate bioequivalence (25). Differences of C_max_ and AUC values were observed in both directions, most probably due to the high variability of the *in vivo* results. Comparing the *in vitro* dissolutions, Asactal dissolved more slowly than all other formulations, which can be seen also with the USP method, but even more typical with the two alternatives of the new method. In case of both RGE and SGE, the onset of dissolution was similar to that of the reference Aspirin Protect formulation, which meets the expectations based on the qualitatively equivalent composition (see Table I.) and similar thickness (see Table V.) of the coatings. The slower rate of dissolution may be explained by the different performance of the tablet cores. As evident from Table I, Asactal contains hydrophobic stearic acid, which may reduce the wettability of the tablet core compared to other formulations. Overall, based on the *in vitro* results, a lower bioavailability compared to the reference product is expected.

In case of ASA Krka, bioequivalence could not be demonstrated in the fasted state. The study showed 16% increase for both AUC and C_max_ compared to the reference product which is consistent with the results of the RGE method, which predicts an earlier release of the ASA Krka formulation compared to Aspirin Protect. The onset of release of ASA Krka obtained from the SGE method is similar to that of Aspirin Protect. However, the slope of its dissolution curve is slightly higher, which generally predicts a higher C_max_ value as well. Based on Table I, the applied plasticizer in the coating of this formulation is triacetin, while the reference product is formulated with triethyl citrate, which may explain the different onset of drug release observed with the RGE method. The slightly higher dissolution rate is probably related to the hydrophilic lactose monohydrate in the tablet core.

In case of Asatrin Teva Protect and ASA Protect Pharmavit there were no clinical data available, thus it was not possible to make *in vitro – in vivo* comparisons. The qualitative compositions of these formulations are equivalent to that of ASA Krka. Accordingly, their dissolution profiles were also similar with the RGE method. Moreover, the onset of their drug release was less affected by the longer acidic pretreatment used in the SGE method. Based on this, both formulations are expected to have higher bioavailability compared to the reference product.

The results also demonstrate the importance of the plasticizer type in the onset of release and the wettability of the tablet core in the rate of dissolution of the tested formulations.

Walgreens Aspirin differs from other formulations tested in the type and thickness of coating, composition of the tablet core, and even in the labeled drug content. This difference occurs especially in case of the SGE method, which indicates that the applied coating material is more sensitive for the longer acidic treatment compared to methacrylic acid−ethyl acrylate 1:1 copolymer. The experienced reduction in the dissolution may be a risk of lower bioavailability in case of subjects with longer gastric residence times.

## Conclusions

Two alternatives of a biorelevant dissolution method – differing in the length of acidic treatment – were successfully developed, modeling the conditions of the stomach and the small intestine in fasted state. Biorelevant molarity and volume of dissolution medium as well as gradual pH change between each tract, were also considered. Six commercially available low-dose enteric coated ASA formulations were tested with the USP method and the two versions of the novel dissolution method.

Despite of the difficulties of demonstrating bioequivalence, all formulations met the acceptance criteria specified in the individual USP monograph of Aspirin Delayed-Release Tablets, which pointed out the importance of an appropriate biorelevant dissolution method. Comparing the compositions of the formulations with the *in vitro* results, the new method, especially with rapid gastric emptying proved to be discriminative. The different plasticizers applied in the coating process appeared to affect the onset of dissolution, while the hydrophilicity of the inactive ingredients affected the dissolution rate by altering the wettability of the tablet cores.

Applying the new method with longer acidic treatment resulted in later onset and slower rate of post-gastric drug release for all formulations. Considering the high variability of *in vivo* gastric residence times, performing the dissolution with both alternatives of the new method may be necessary to lower the risk of bioinequivalence of similar generic drug candidates.

Based on the relationship between the *in vitro* dissolution and the limited available bioequivalence data, and the increased discriminating power of the new dissolution method, an enhanced *in vivo* predictivity can also be assumed.

Overall, we conclude that the new method can be a good alternative for reaching a better understanding of the post-gastric behavior of enteric-coated formulations which is essential to get appropriate information on intestinal release and bioavailability.

## Abbreviations

ACN: Acetonitrile
ASA: Acetylsalicylic acid
API: Active Pharmaceutical Ingredient
BA: Bioavailability
BE: Bioequivalence
CI: Confidence interval
EC: Enteric coating
EMA: European Medicines Agency
GI: Gastrointestinal
HPLC: High Performance Liquid Chromatography
MMC: Migrating motor complex
NSAID: Nonsteroidal anti-inflammatory drug
PVAP: Polyvinyl acetate-phthalate
QC: Quality control
RGE: Rapid gastric emptying
SGE: Slow gastric emptying
AUC: The area under the plot of plasma concentration of a drug *versus* time after dosage
C_max_: The maximum concentration that a drug achieves in a specified compartment of the body after administration
t_max_: The time it takes a drug to reach the maximum concentration in plasma
USP: United States Pharmacopoeia
FDA: U.S. Food and Drug Administration
